# Incorporating public priorities in the Ocean Health Index: Canada as a case study

**DOI:** 10.1371/journal.pone.0178044

**Published:** 2017-05-24

**Authors:** Rémi M. Daigle, Philippe Archambault, Benjamin S. Halpern, Julia S. Stewart Lowndes, Isabelle M. Côté

**Affiliations:** 1 Institut des Sciences de la Mer, Université du Québec à Rimouski, Rimouski, Québec, Canada; 2 National Center for Ecological Analysis and Synthesis, University of California, Santa Barbara, Santa Barbara, California, United States of America; 3 Department of Biological Sciences, Simon Fraser University, Burnaby, British Columbia, Canada; 4 Département de Biologie, Laval University, Québec, Canada; 5 Hopkins Marine Station, Stanford University, Stanford, California, United States of America; 6 Silwood Park, Imperial College London, Ascot, United Kingdom; 7 Bren School of Environmental Science & Management, University of California, Santa Barbara, Santa Barbara, California, United States of America; Universidade de Aveiro, PORTUGAL

## Abstract

The Ocean Health Index (OHI) is a framework to assess ocean health by considering many benefits (called ‘goals’) provided by the ocean provides to humans, such as food provision, tourism opportunities, and coastal protection. The OHI framework can be used to assess marine areas at global or regional scales, but how various OHI goals should be weighted to reflect priorities at those scales remains unclear. In this study, we adapted the framework in two ways for application to Canada as a case study. First, we customized the OHI goals to create a national Canadian Ocean Health Index (COHI). In particular, we altered the list of iconic species assessed, added methane clathrates and subsea permafrost as carbon storage habitats, and developed a new goal, 'Aboriginal Needs', to measure access of Aboriginal people to traditional marine hunting and fishing grounds. Second, we evaluated various goal weighting schemes based on preferences elicited from the general public in online surveys. We quantified these public preferences in three ways: using Likert scores, simple ranks from a best-worst choice experiment, and model coefficients from the analysis of elicited choice experiment. The latter provided the clearest statistical discrimination among goals, and we recommend their use because they can more accurately reflect both public opinion and the trade-offs faced by policy-makers. This initial iteration of the COHI can be used as a baseline against which future COHI scores can be compared, and could potentially be used as a management tool to prioritise actions on a national scale and predict public support for these actions given that the goal weights are based on public priorities.

## Introduction

The well-being of people and the health of the oceans are inextricably linked [1,2]. However, measuring ocean health has been a long-standing challenge [3,4]. A multitude of indicators have been used, each of which measures relatively narrow aspects of ocean condition that are relevant to specific management objectives, such as maintaining biodiversity or the integrity of marine communities, minimising the endangerment of species or loss of habitat, limiting human influence, safeguarding human health, or delivering one of the countless goods and services provided by the sea [5–7]. Arguably, oceans in a desirable (‘healthy’) state should be able to deliver all of these goals, hence a useful index of ocean health should be able to capture the widely disparate aspects measured by multiple indicators [4,8]. The challenge of combining indicators potentially expressed in different units, measured on different scales, or that are fundamentally different (e.g., quantitative vs. qualitative) is high, but this is a necessary task if we are to move beyond simply equating ocean health with the extent to which the marine environment has been degraded by human activity [9].

Recently, Halpern et al. [8,10] proposed the Ocean Health Index (OHI), a composite metric that comprises 10 public goals for healthy and sustainable coupled human and natural systems. The OHI is different from many other previous attempts at measuring ocean health because it focuses on the sustainable delivery of a suite of ecosystem benefits to people rather than on individual benefits separately or on activities that damage ocean integrity. The goals range from concrete benefits, such as food provision, which is a measure of how much food comes from wild-caught fishing and mariculture relative to a sustainable optimum, to seemingly less tangible values, such as a sense of place, which is represented by the status of identified special places and iconic species. The method combines disparate measurements into a single index score, which can be tracked over time to reflect trends in overall ocean health. This single score is composed of scores calculated for each goal: goal scores are based on a combination of current status and likely future status, which itself depends on trends, the strength of current pressures and scope for resilience (factors that affect a goal positively; e.g. ecological integrity) [8]. Most importantly, from a management perspective, the performance of each goal is assessed in relation to reference points that are realistic targets for sustainability rather than to a pristine state [11]. By comparing the performance of the different goals and their respective contributions to the overall OHI score, the index can potentially be used to identify which goal(s) may benefit from management intervention.

While the OHI framework was first used to assess all coastal nations and territories globally [11], analyses at such a scale are too coarse to guide specific interventions at the municipal, regional, sub-national or national levels, which are the scales at which many decisions are made. A strength of the OHI is that it can be tailored to accommodate data sources available at different resolutions for finer-scale analyses, and adapted to the goals and specific targets (i.e., reference points) of any given location [12]. Several national and regional OHI assessments have been completed, such as for the west coast of the USA [13], Brazil [14], and Fiji [15]. In each case, the analyses used higher-resolution data when available, place-specific reference points, and regional proxies for calculating each of the goals relevant to the assessment, as originally defined by Halpern et al. [8]. Halpern et al. [13] also demonstrated how OHI assessments can be used to explore the consequences of past and hypothetical future management interventions, as well as to reconstruct historical trends for goals for which time-series data exist.

Assessment of ocean health at national and subnational levels can be achieved by using nation- or state-specific data [13,14]. However, such analyses also present an opportunity to reflect national values and other spatially specific conditions in a way that a global analysis cannot capture. Different cultures value different aspects of the oceans, their resources and biodiversity, which can be reflected in OHI assessments by weighting different goals in relation to the importance accorded to them by people. For example, the OHI assessment of the west coast of the USA, Halpern et al. [13] used a multi-criteria decision-making approach to elicit a ranking of OHI goals from expert stakeholders with specific interests in various ocean sectors (e.g., fisheries, conservation NGOs, etc.). Surprisingly, the weights elicited were relatively similar across most goals, although the weights for the goals of Clean Water and Sense of Place were 3–4 times higher than the rest [16]. The weighted averaging of goals resulted in higher overall OHI scores in some regions and lower scores in others [13]. It is not clear, however, how the values elicited by experts with vested interests in certain goals reflect the values held by the population at large.

The aim of the present study was to evaluate how the OHI framework could be tailored for Canada’s oceans. We adapted the global OHI assessment to reflect Canadian priorities and values by evaluating Canada’s oceans collectively. While Canada has vast territorial waters and exclusive economic zone, we have chosen to evaluate Canada’s 3 oceans in one assessment to provide a common framework as a basis for future higher resolution sub-national OHI assessments. We did not treat each ocean as separate regions since doing so would require specific regional data that are better suited for the sub-national scale assessments. We carefully evaluated the 10 goals defined in the global OHI and asked how well they reflected the Canadian context (Table 1). In particular, we explored how to incorporate the extent to which Canadian Aboriginal people have access to fishing and hunting grounds–a right that is enshrined in the Canadian constitution (www.justice.gc.ca/eng/csj-sjc/just/05.html). To reflect the values of the Canadian public at large, we designed a customized online survey to elicit goal preferences, and distributed it in a stratified manner across Canadian provinces. Answers to this survey generated both an absolute quantitative score and a relative ranking for each goal [17], and included demographic information. The objectives of this study were (1) to produce a quantitative estimate of the health of Canadian oceans to be used as a baseline for future assessments, (2) to examine the effect of various methods of goal weighting on the Canadian OHI (COHI), and (3) to describe variation in COHI scores arising from regional political, and age-related variation in goal rankings across Canada.

**Table 1:** Definitions of goals of the Canadian Ocean Health Index provided to survey respondents.

Goal	Definition
Food Provision	The amount of fish and seafood we can extract sustainably from our oceans.
Aboriginal Needs	The extent to which Canada’s Aboriginals are able to access ocean resources for subsistence, social and ceremonial purposes.
Natural Products	The amount of non-food products we can harvest sustainably from our oceans to make, for example, pharmaceutical products, fertilisers, jewellery, etc.
Carbon Storage	The extent of coastal habitats, like seagrass beds and marshes, we have that help remove atmospheric carbon that would otherwise contribute to climate change
Coastal Protection	The extent of coastal habitats, like kelp and seagrass beds, we have to break wave action and protect coasts during storms
Coastal Livelihoods	The number of people employed in and the revenue generated from marine-related industries (such as fishing and tourism)
Tourism & Recreation	The number of people (both local and tourists) that take part in recreational activities on the coast
Iconic Places & Species*	The health of and level of protection given to species and places that are particularly special to Canadians
Clean Waters	The cleanliness of our coastal waters, that is, how free of trash, chemicals, disease, agricultural effluent they are
Biodiversity	The extent to which the variety of marine life in Canada is being maintained

* The ‘Sense of Place’ goal was simplified to ‘Iconic Places & Species’ (*i*.*e*. the combined names of the subgoals) for the survey.

## Methods

Our methods and data sources were based on those of Halpern et al. [8] and modified as described below to improve the quality and relevance of the data and to address specific Canadian issues (Table 2). These modifications involved data substitutions and additions, the definition of a new “Aboriginal Needs” goal that replaces the OHI’s “Artisanal Fishing Opportunities” goal, and the use of goal weightings, derived from a web-based survey [17], to quantify the relative importance of each goal to Canadians.

**Table 2:** New data layers used in the calculation of the Canadian Ocean Health Index.

Data Layer	Brief Description	Goals	Dimension	Start Year	End Year	Native Resolution	Reference
CIW	Canadian Index of Wellbeing	All	Pressure, Resilience	1994	2010	Annual	[18]
GDP	Canadian Gross Domestic Product relative to 1994	All	Pressure, Resilience	1994	2010	Annual	[18]
Clathrates	Estimation of area likely to contain methane clathrates based on depth	CS	Status	2015	2015	1 min	[19]
Permafrost	Estimation of area which is likely to contain sub-sea permafrost based on depth and latitude	CS	Status	2015	2015	1 min	[19–21]
Global CO_2_	Globally averaged marine surface annual mean CO_2_ data	CS	Trend	1980	2013	Annual	[22]
New Species List	Species that appear on Canadian currency	ICO	Status	2015	2015	Species	[23]
COSEWIC status	Current status recommended by COSEWIC	ICO	Status	2015	2015	Species	[24]
RFNB	Revised Northern Food Basket	AN	Status, Trend	2005	2010	Community	[25]
CPI-Food	Consumer Price Index for “Food” by urban centre	AN	Status, Trend	1914	2013	Community	[26]
Aboriginal Communities	Location and population living in First Nation and Inuit communities	AN	Status	2013	2013	Community	[27,28]
Gas prices	Gasoline and fuel oil, average retail prices by urban centre (Regular unleaded gasoline at self service filling stations)	AN	Status, Trend	1979	2014	Community	[29]
Sea-ice extent	Consistent, up-to-date sea ice extent and concentration images and monthly data values.	AN	Status, Trend	1979	2013	25 km	[30]

Goals include Aboriginal Needs (AN); Carbon Storage (CS); Iconic species (ICO) subgoal which is part of Sense of Place.

As for all past OHI assessments, we defined a healthy ocean as one that sustainably delivers a range of benefits to people. The OHI is not a measure of ocean pristineness but explicitly includes human use so that OHI scores can be used, among other things, for management purposes. Whether a health index should reflect pristineness or sustainable use has been discussed elsewhere (e.g., [8]), including a review of reports and documents identifying humans as an integral part of ecosystems in a policy context (see [13] supplemental information). We considered the OHI framework most appropriate because it has a human perspective, with reference points set to be Specific, Measurable, Achievable, Realistic, and Time-bound (i.e., ‘SMART’; [11]).

The code for data wrangling [31] and analysis [32] related to this manuscript have been archived on Zenodo.

### The OHI framework

The OHI comprises 10 widely accepted public goals, many of which represent ecosystem services delivered by the oceans (Table 1). An index score for each goal, I_i_, is calculated as an average of this goal’s current status x_i_ and its near-term future status ${\hat{x}}_{\text{i},\text{F}}$. Current status x_i_ is expressed as the ratio of the present status value of a goal, X_i_, over a reference point, X_i,R_, which is identified as a desirable, sustainable status value for that goal and scaled from 0 to 100. Halpern et al. [8] and Samhouri et al. [11] provide explicit guidelines for setting reference points.

Near-term future status ${\hat{x}}_{\text{i},\text{F}}$ is given as:

$${\hat{x}}_{i,F}=\;{\left(1\;+\;\delta \right)}^{-1}\;\left[1\;+\;\beta T_{i}\;+\;\left(1-\beta \right)\left(r_{i}-p_{i}\right)\right]x_{i}$$

where δ is a discounting rate, set to 0 in this case because of the short timeframe, r_i_ is resilience, p_i_ is pressures, and T_i_ is the recent (~ 5 years) trend. We followed [8] in setting the weighting factor β to 0.67 to reflect the fact that the recent trend is a better indicator of the likely trajectory of the goal status in the near future than the resilience and pressure terms. The recent trend T_i_ is calculated as the slope of the change in status of the goal over the past 5 years.

Resilience for each goal (r_i_) represent factors that affect a goal positively, and incorporates ecological integrity (ϒ_E_; e.g., the. relative condition of an assessed species), regulations aimed at mitigating ecological pressures relevant to that goal (G; e.g.,. regulations for sustainable mariculture practices), and social integrity (ϒ_S_; i.e., the processes internal to a human community that affect its resilience). These three aspects of resilience are scaled from 0 to 100 and combined as:

$$r_{i}=\;\gamma \left(\frac{Y_{E}+G}{2}\right)+\left(1-\gamma \right)Y_{S}$$

where the weighting factor γ is set to 0.5 since the ecological and social integrity components are deemed to be equally important.

The pressures for each goal (p_i_) represent factors that affect a goal negatively, and comprise ecological (p_E_) and social pressures (p_S_) such that:

$$p_{i}\;=\;\gamma \;p_{E}\;+\;\left(1-\;\gamma \right)\;p_{S}$$

In the absence of justifying evidence, the weighting factor γ is set to 0.5 [8]. Ecological pressures include five broad categories: fishing pressure, habitat destruction, climate change, water pollution and species introductions. Social pressures includes six components: control of corruption, government effectiveness, political stability, regulatory quality, rule of law, voice and accountability from the Worldwide Governance Indicators (WGI), that are combined into a social pressure score [8].

Finally, the overall index (I) score is calculated as the weighted sum of the 10 goal-specific scores (I_i_) divided by the sum of the maximum possible values for each goal:

$$I\;=\;\frac{\sum_{i=1}^{N}\alpha_{i}I_{i}}{\sum_{i=1}^{N}\alpha_{i}x_{i}^{max}}\;\times \;100$$

where the sum of the goal-specific weights α_i_ equals 1. The overall OHI is therefore expressed as a percentage.

### Data substitutions

We recalculated Canada’s OHI scores from the global study (i.e., at a national level) after making some modifications to better represent Canada. More specifically, we substituted data underlying social pressure and resilience, the habitats included in the Carbon Storage goal, the species included in the Iconic Species subgoal, and the definition and data underlying the Aboriginal Opportunities goal. These are all described in more detail in the paragraphs below.

#### All goals: Social pressure and resilience

As a Canadian alternative to the Worldwide Governance Indicators (WGI) used in the original OHI [8], we used the Canadian Index of Wellbeing (CIW) as a measure of social pressure and resilience [18]. The CIW is a composite index that measures eight quality of life categories: Community Vitality, Democratic Engagement, Education, Environment, Healthy Populations, Leisure and Culture, Living Standards, and Time Use. We chose to use the CIW instead of the WGI because (1) it is a ‘Canadian’ approach that is not limited by global data availability, and (2) it includes dimensions other than governance that affect social pressures and resilience (e.g., Education).

The CIW is normally interpreted relative to its initial value, calculated in 1994. Moreover, it is usually expressed in relation to gross domestic product (GDP) to track whether gains in quality of life mirror economic growth. In the COHI, we used the ratio of CIW:GDP × 100 from 2013 and scaled it to 1994 levels, so that a value of 100 indicates a GDP to CIW ratio as it was in 1994. Levels below 100 mean that the GDP has increased without a corresponding increase in CIW, or that the CIW has decreased.

#### Carbon Storage goal: Current status and recent trend

**Fig 1:**
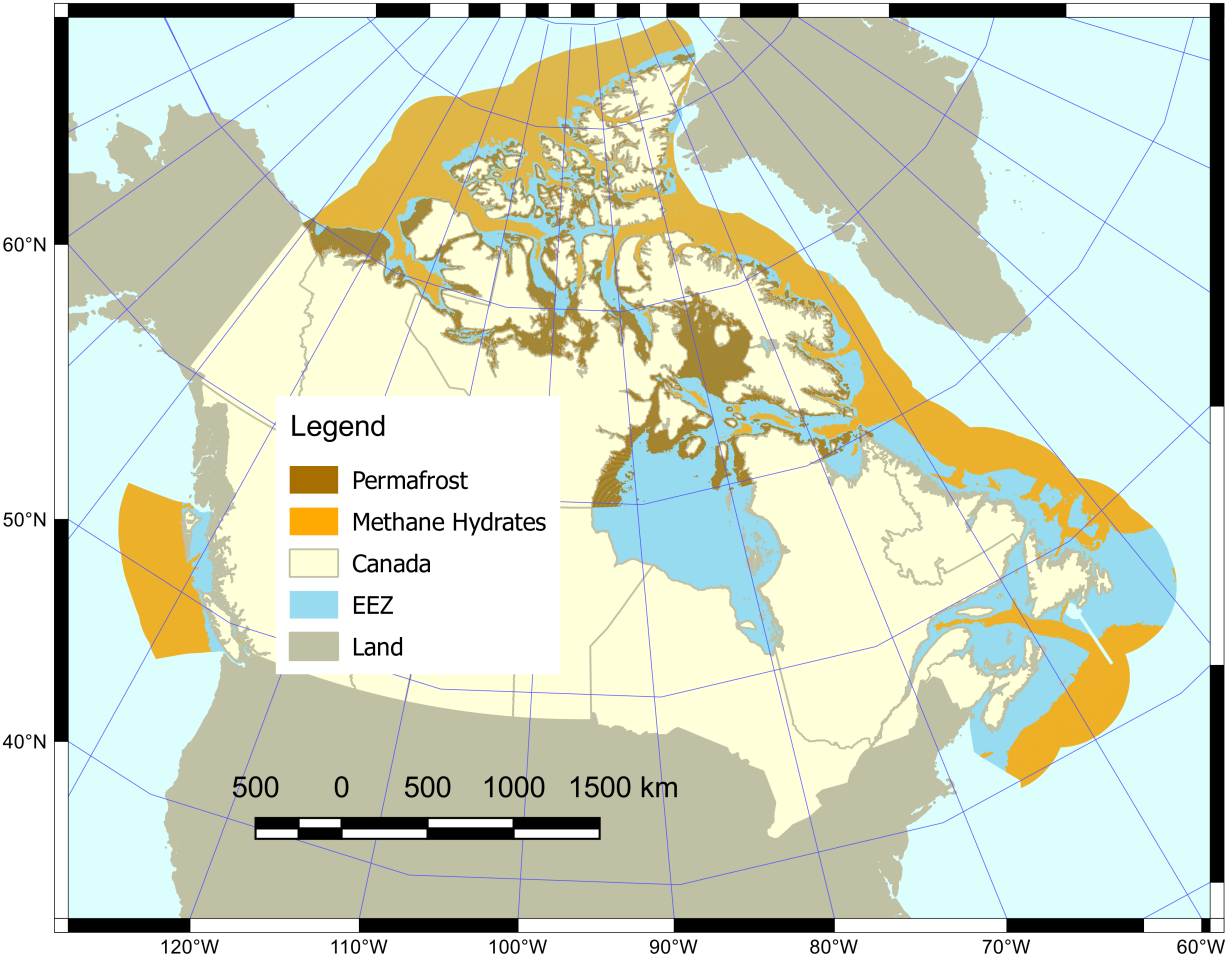
Map of carbon storage areas in Canada’s oceans. These areas are delimited by depth boundaries that represent where each ‘habitat’ type may be found. Subsea permafrost is found at depths of 0–120 m, north of 60°N, and methane clathrates are found below 300 m.

The coastal habitats contributing to Canada’s Carbon Storage goal in Halpern et al. [8] included seagrasses and salt marshes. However, in Canada’s Arctic region a large amount of carbon is also stored in decaying organic material within and below subsea permafrost (<120 m deep) and as methane clathrates in deeper waters (>300 m) [20,33,34]. We therefore added these as ‘habitats’ to the Canadian Carbon Storage goal. The current status of the subsea permafrost and methane clathrate habitats was measured as the current areal extent of these habitats relative to a reference extent (Fig 1). Since subsea permafrost is usually found to a depth of 120 m [20,21], we assumed that all benthic area between 0 and 120 m that lies north of 60°N potentially contains permafrost. Similarly, we assumed that all benthic area below 300 m potentially contains methane clathrates since these compounds only form under specific temperature–pressure combinations that are common below 300 m [33]. Areal extents were obtained by measuring the area covered by the corresponding depths limits using the ‘marmap’ R package [19].

To quantify recent trends in subsea permafrost and methane clathrate habitats, we used sea surface temperature anomaly relative to pre-industrial levels. An ocean temperature increase of 3°C from 2005 levels is predicted to result in destabilization of most (84%) oceanic gas hydrate deposits and release of 4200 Gt of carbon [35]. We therefore defined the condition of the methane clathrate and subsea permafrost ‘habitats’ as equivalent to the northern hemisphere sea surface temperature anomaly above pre-industrial levels scaled to 3.65°C, which corresponds to a 3°C increase from 2005 levels [22]. That is to say, an anomaly of 3.65°C corresponds to a habitat health of 0, and a smaller increase of 2.15°C would correspond to a habitat health of 0.5. Recent trend was calculated from the 5 most recent years in the globally averaged, marine surface annual mean CO_2_ data [22]. We used the same pressures and resilience as those defined for the Carbon Storage goal in Halpern et al. [8]. We incorporated these new components of the Carbon Storage goal by weighting by proportional area covered by these habitats.

#### Iconic species subgoal: Current status and recent trend

To identify iconic marine species, Halpern et al. [8] used the World Wildlife Fund’s global and regional lists for Priority Species (i.e., those species that are especially important to people for their health, livelihoods, and/or culture) and Flagship Species (i.e., ‘charismatic’ and/or well-known). From these lists, the species considered to be iconic to Canada included polar bear, eight whale species, and five species of sharks.

We revised and broadened this list by considering instead marine wildlife that has featured on Canadian coins (both circulation and collector), because the products of national mints generally celebrate people, events, places and wildlife of national significance. A search of the database of the Royal Canadian Mint (www.mint.ca) yielded 19 marine species, only four of which were used in Halpern et al. [8] (Table 3). The revised list includes six mammals, two birds, nine bony fishes and two invertebrate species (Table 3).

**Table 3:** Species identified on Royal Canadian Mint currency.

Common name	Scientific name
Lobster	*Homarus americanus*
Leather star	*Dermasterias imbricata*
Atlantic Cod	*Gadus morhua*
Chum Salmon*	*Oncorhynchus keta*
Coho Salmon*	*Oncorhynchus kisutch*
Pink Salmon*	*Oncorhynchus gorbuscha*
Rainbow Trout	*Oncorhynchus mykiss*
Sockeye Salmon	*Oncorhynchus nerka*
Chinook Salmon*	*Oncorhynchus tshawytscha*
Atlantic Salmon	*Salmo salar*
Arctic Char	*Salvelinus alpinus*
Orca	*Orcinus orca*
Humpback◊	*Megaptera novaeangliae*
Bowhead◊	*Balaena mysticetus*
Blue whale◊	*Balaenoptera musculus*
Beluga	*Delphinapterus leucas*
Polar Bear◊	*Ursus maritimus*
Common Loon	*Gavia immer*
Bald Eagle	*Haliaeetus leucocephalus*

* These salmon species were added because there was a non-specific reference to salmon.

^◊^ denote species used in the original OHI (Halpern et al. 2012).

The current status and recent trends for each of the iconic species were obtained from the most recent assessment by the Committee on the Status of Endangered Species in Canada (COSEWIC). The spatial coverage weight was calculated based on the ranges identified by COSEWIC and the risk weights were modified from those in Halpern et al. [8] to match the COSEWIC statuses (Table 4).

**Table 4:** Risk weight for each COSEWIC status.

COSEWIC status	Risk weight
Not Assessed	0
Non-active	0
Data Deficient	0
Not at Risk	0
Special Concern	0.2
Threatened	0.4
Endangered	0.6
Extinct	1
Extirpated	1

### New goal: Aboriginal Needs

The original OHI goal of ‘Artisanal Fishing Opportunities’ meant to reflect the ability to conduct sustainable, artisanal-scale fishing when the need is present. Artisanal fishing was defined as fisheries involving households, cooperatives or small firms that use a small amount of capital and energy and small fishing vessels (if any), make relatively short fishing trips, and use fish mainly for local consumption or trade [8]. Artisanal fishing may happen under a commercial license (e.g., a family-run boat or individual shellfish harvesting permit), or under a recreational fishing permit (e.g., families fishing with rods for fish to eat). Importantly, this goal is about the opportunities to carry out such activities rather than about the quantity of food they provide.

While several forms of artisanal fishing occur in Canada, it is most prevalent among Aboriginal communities. Indeed, the Canadian constitution has enshrined the right of Canadian Aboriginal people to fish and hunt for food, social, and ceremonial purposes, which takes precedence over commercial and other interest [36]. Our new goal ‘Aboriginal Needs’, which replaces the ‘Artisanal Fishing Opportunities’ goal, represents the extent to which Canada’s Aboriginals are able to access ocean resources for subsistence, social, and ceremonial purposes. This goal is based on the physical access to the resources, and the financial factors that determine how many individuals participate in a traditional hunt or fisheries.

Ice cover was identified as a critical variable in limiting physical access to traditional hunting and fishing grounds for Canadian Aboriginals [37,38]. Current status and recent trend in ‘Aboriginal Needs’ were therefore estimated by quantifying the areal extent of sea ice in a 300 km radius of each Aboriginal community using the National Snow & Ice Data Center Sea Ice Index [30].

The primary financial factors that determine Aboriginal access to natural resources are related to the cost of participating in a traditional hunt or fisheries relative to the price of purchasing food (Vincent L’Hérault from ARCTIConnexion (http://arcticonnexion.ca/en/) and Joey Angnatok, pers. comm.). The cost of participation was based on the price of fuel [29] for snowmobiles, boats, and all-terrain vehicles. The estimated price of food for Inuit communities was based on the Revised Northern Food Basket (RNFB), which quantifies the price of purchasing a nutritious diet for a family of four in northern communities [25]. Unfortunately, the RNFB was not available for all communities (44 of 356), so the estimated price *F*_*i*_, in Canadian dollars, of the Revised Northern Food Basket for community *i* was estimated using the equation from a linear regression (parameters in Table 5) of existing data as:

$$F_{i}=\;a+b_{1}RFNB+b_{2}RFNB\_dist+b_{3}ICCPI+b_{4}ICCPI\_dist+b_{5}Pop$$

where *RNFB* is the value of RFNB for the nearest available community; *RFNB_dist* is the distance to the nearest community; *ICCPI* is the value of the food Inter-Community Consumer Price Index for the nearest major city, which we used as a proxy for food prices at the point of distribution; *ICCPI_dist* is the distance to the nearest major city; and *Pop* is the population of community *i*.

**Table 5:** Model parameters (± standard error) for the calculation of the estimated price (Canadian dollars) of the Revised Northern Food Basket (see Equation 1 in Methods).

	Inuit		First Nations	
Intercept	1262	±423	1606	±413
*RNFB*	0.625	±0.239	NA	
*RFNB_dist*	0.035	±0.017	NA	
*ICCPI*	-10.89	±4.01	-12.86	±4.05
*ICCPI_dist*	0.024	±0.018	0.048	±0.011
*Pop*	-0.007	±0.004	-0.006	±0.004
Adj. R^2^	0.49		0.42	
F	9.13		11.51	
p	< 0.001		< 0.001	

The model parameters of *RNFB* (Revised Northern Food Basket) and *RFNB_dist* (distance to nearest community) were not used for First Nations communities since they did not improve model fit. *ICCPI* is the value of the food Inter-Community Consumer Price Index for the nearest major city, which we used as a proxy for food prices at the point of distribution; *ICCPI*_dist is the distance to the nearest major city; and *Pop* is the population size.

The Aboriginal Needs (AN) index is a population-weighted mean of the AN score for each Aboriginal community and is calculated as:

$$AN=\;\overset{n}{\underset{i=1}{\sum }}\frac{P_{i}}{P_{tot}}\times \frac{\frac{F_{i}}{G_{i}}}{\frac{Fb_{i}}{Gb_{i}}}\;\times \frac{I_{i}}{Ib_{i}}$$

where *P*_*i*_ and *P*_*tot*_ are the population in community *i* and the total population summed across all communities, respectively; *G*_*i*_ is the price of gasoline (Canadian cents per litre) in the nearest major city [39]; *I*_*i*_ is the average yearly percent ice cover within a 300-km radius around community *i*; and, *Fb*_*i*_, *Gb*_*i*_ and *Ib*_*i*_ are the 1979 baseline levels for food, gasoline and ice cover, respectively. The AN is therefore set to 1 in 1979 and decreases as ice cover decreases and/or the price of gasoline increases relative to the cost of the Revised Northern Food Basket. If *I*_*i*_ was 0 for an entire time series, *I*_*i*_ ÷ *Ib*_*i*_ was set to 1. In these cases, there was no decrease in availability of ice cover for hunting and fishing near those Aboriginal communities; therefore, there was no decrease in AN due to ice.

### Goal weighting

Weightings of the 10 ocean health goals were derived from a broad survey of Canadian attitudes and preferences towards ocean-derived benefits. Detailed survey methods and findings are reported in Daigle et al. [17]. In brief, to evaluate the public perception of benefits provided by the ocean, we used a market research company (ResearchNow; www.researchnow.com) to distribute an online survey to a representative cross-section of Canadians (n = 2026 respondents) stratified by region (British Columbia, Prairies, Ontario, Quebec, Atlantic). The survey did not sample the northern territories since there were not enough potential respondents in the database to include this region. The study was approved as ‘minimal risk’ by the Research Ethics Board at Simon Fraser University (Study Number: 2013s0895). Our survey requested information on the perceived importance of the 10 goals used in the present study (Table 1). Respondents were first asked to read the definition of each goal as part of a learning exercise and rate the importance of that goal on a Likert scale (1 - “Not Important” to 5 - “Very Important”). The respondents then completed a best/worst choice experiment. To generate the overall utility (i.e., importance) of each goal across all respondents, we fitted a discrete choice (logit) model with one latent class in LatentGOLD Choice (v.5.0).

To evaluate the relative effectiveness of different weighting techniques, we used four methods to weight the ocean health goals: ‘Equal’, ‘Likert’, ‘BW-Rank’, and ‘BW-DCE’. The ‘Equal’ method weights all goals equally (weight = 1). This method is the ‘status quo’ and requires no survey or data analysis. The ‘Likert’ method weights the goals based on the Likert scores, by averaging importance score of each goal across respondents. This method is the simplest weighting method both in terms of survey design and analysis. We also derived two different weightings from the answers to the best/worst choice questions. The best/worst scaling discrete choice survey questions are more intensive to design. These questions can be analysed using two methods of varying difficulty. The ‘BW-Rank’ method simply used the inverse ranks of respondents’ answers as weights (i.e., so first-ranked goal carried the largest weight), and the ‘BW-DCE’ method used as weights the discrete choice model coefficients, which represent the probability of choosing one goal over another, from the LatentGOLD analysis. To incorporate these raw values into weights that can be used with the OHI, we transformed the raw mean Likert scores, ranks, or coefficients to a scale appropriate for weighting. We added a constant to all values so that the lowest value was equal to 1. We then divided the adjusted values by another constant so their sum would equal 10. In all cases, subgoals weights are 0.5, except for Food Provision where subgoals were weighted relative to yield.

The survey also generated demographic information, so we were able to compare weightings between regions (British Columbia, Prairies, Ontario, Quebec, Atlantic), and respondents of different age groups (20 to 24, 25 to 34, 35 to 44, 45 to 54, 55 to 64, and > 65 years old), political affiliations (New Democratic Party or Green Party of Canada, Liberal Party of Canada, Conservative Party of Canada, and other affiliations), and levels of environmental engagement (i.e., members of environmental NGOs vs. non-members). These factors were previously shown to be associated with differences in importance accorded to different ocean-related benefits [17].

## Results

### Aboriginal Needs

**Fig 2:**
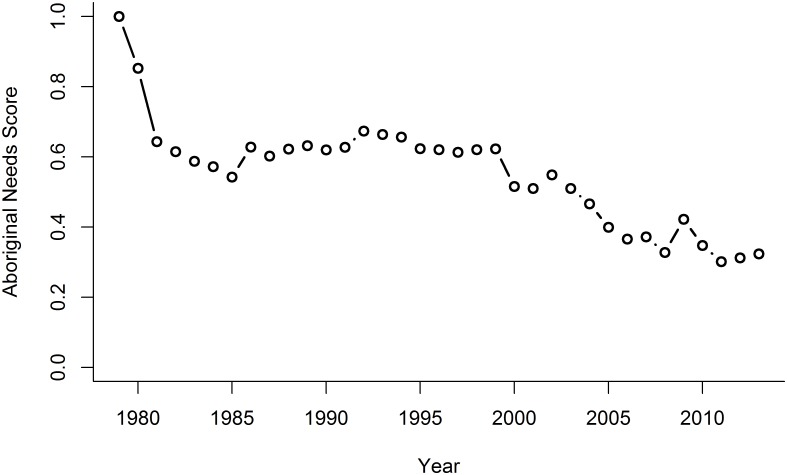
Trajectory of Aboriginal Needs current status over time. The Aboriginal Needs goal includes the extent of sea ice and the cost of participating in traditional hunting or fishing (i.e., gasoline price) relative to the cost of purchasing food.

The Aboriginal Needs (AN) goal score decreased precipitously from 1979, the base year, to 1981. It then remained fairly stable until 1999, which marked the onset of a steady decline (Fig 2). In 2013, only 25 of 357 Aboriginal communities had less than 90% of the baseline (1979) ice cover, and only 5 communities had less than 50% of baseline ice cover. Therefore, a large proportion of the decrease in the current status of the AN goal can be attributed to an increase in the price of fuel relative to the price of food rather than to a change in ice cover. Since 1979, fuel prices have increased ~550% while food prices have increased ~300%. For 2013, the relative increase in fuel:food price ratio by itself decreased the AN score to 54, while decreases in ice cover lowered it further to 32 (Table 6).

**Table 6:** The values included in the calculation of the Canadian Ocean Health Index.

Goal	Subgoal	Score	Status	Future	Trend	Pressures	Resilience
Food Provision		64.28	58.66	69.90	0.02	NA	NA
Food Provision	FIS	59.36	54.00	64.72	0.02	16.69	72.81
Food Provision	MAR	92.88	85.77	100.00	0.04	14.01	68.68
Aboriginal Needs		35.42	32.39	38.45	-0.02	13.58	73.66
Natural Products		44.02	41.90	46.13	-0.16	13.81	76.43
Carbon Storage		61.64	57.28	66.00	-0.01	16.52	64.06
Coastal Protection		95.89	91.79	100.00	-0.01	23.81	64.06
Coastal Livelihoods		89.87	86.74	93.00	0.03	NA	NA
Coastal Livelihoods	LIV	79.74	73.48	86.00	0.03	14.89	60.08
Coastal Livelihoods	ECO	100.00	100.00	100.00	0.02	14.87	73.55
Tourism & Recreation		25.19	23.93	26.45	0.00	30.64	62.97
Iconic Places & Species		84.91	80.00	89.81	-0.10	16.99	74.46
Iconic Places & Species	LSP	33.89	25.27	42.52	0.78	22.84	72.06
Iconic Places & Species	SP	59.40	52.63	66.17	0.34	NA	NA
Clean Waters		78.70	84.31	73.09	-0.32	38.19	62.97
Biodiversity		91.05	84.83	97.28	-0.04	NA	NA
Biodiversity	HAB	92.72	85.43	100.00	0.00	14.73	75.38
Biodiversity	SPP	89.39	84.22	94.55	-0.08	19.67	72.83
OHI		64.55	NA	67.65	NA	NA	NA

Suboals include Wild Caught Fisheries (FIS); Mariculture (MAR); Livelihoods (LIV); Economies (ECO); Lasting Special Places (LSP); Iconic Species (SP); Habitats (HAB); Species (SPP). Score, current status (a goal’s current value compared to its reference point), likely future state (indicator of what the status score is likely to be in five years), trend (average percent change of a goal’s status over the most recent five years), pressure (ecological and social factors that decrease status) and resilience (ecological factors and social initiatives that increase status by reducing or eliminating pressures) of the goals and subgoals

### Canadian-izing the Ocean Health Index

**Fig 3:**
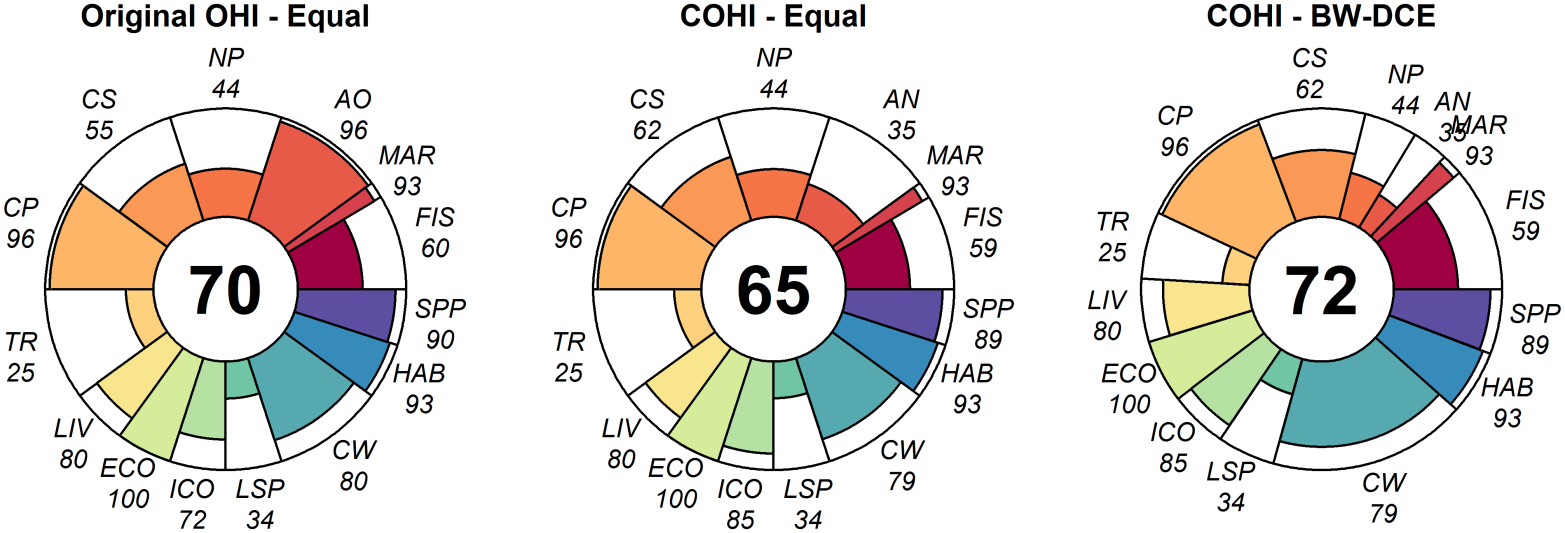
Original Ocean Health Index (OHI) and Canadian OHI (COHI) scores, for individual goals or subgoals (coloured petals) and overall (central number) for 2013. In COHI–Equal, all goals have equal weighting; in COHI–BW-DCE, goals are weighted based on a latent-class discrete choice model coefficients. Numbers in italics show goal current status. Mariculture (MAR) and Fisheries (FIS) subgoals are part of the Food Provision goal; Aboriginal Needs (AN); Natural Products (NP); Carbon Storage (CS); Coastal Protection (CP); Tourism & Recreation (TR); Livelihoods (LIV) and Economies (ECO) subgoals are part of the Coastal Livelihoods goal; Iconic species (ICO) and Lasting Special Places (LSP) subgoals are part of Sense of Place; Clean Waters (CW); Habitat (HAB) and Species Protection (SPP) subgoals are part of the Biodiversity goal.

With equal goal weighting, modifying the OHI data sources and transforming the Artisanal Fishing Opportunities into the Aboriginal Needs goal decreased the Canadian OHI (COHI) score to 65 compared to the original OHI score of 70 (Fig 3). Most of the decrease is attributable to the score of AN (score = 35), which is much lower than that of the Artisanal Fishing Opportunities (AO) goal in the original OHI (score = 96; Fig 3). However, the scores for Iconic Species and Carbon Storage, which were both altered by large data additions, were higher post-modification (Fig 3). The addition of new iconic species made the score for this subgoal increase from 72 to 85. Similarly, the addition of carbon-rich sediments as a ‘habitat’ type in northern Canada increased the score for Carbon Storage from 55 to 62.

The scores for some goals and subgoals (e.g. Fisheries, Clean Waters), for which the status and trends data sources remained the same, declined slightly. These small differences were caused by changes in values of pressures and resilience related to the use of the more relevant Canadian Index of Wellbeing.

### Weighting the Canadian Ocean Health Index

**Fig 4:**
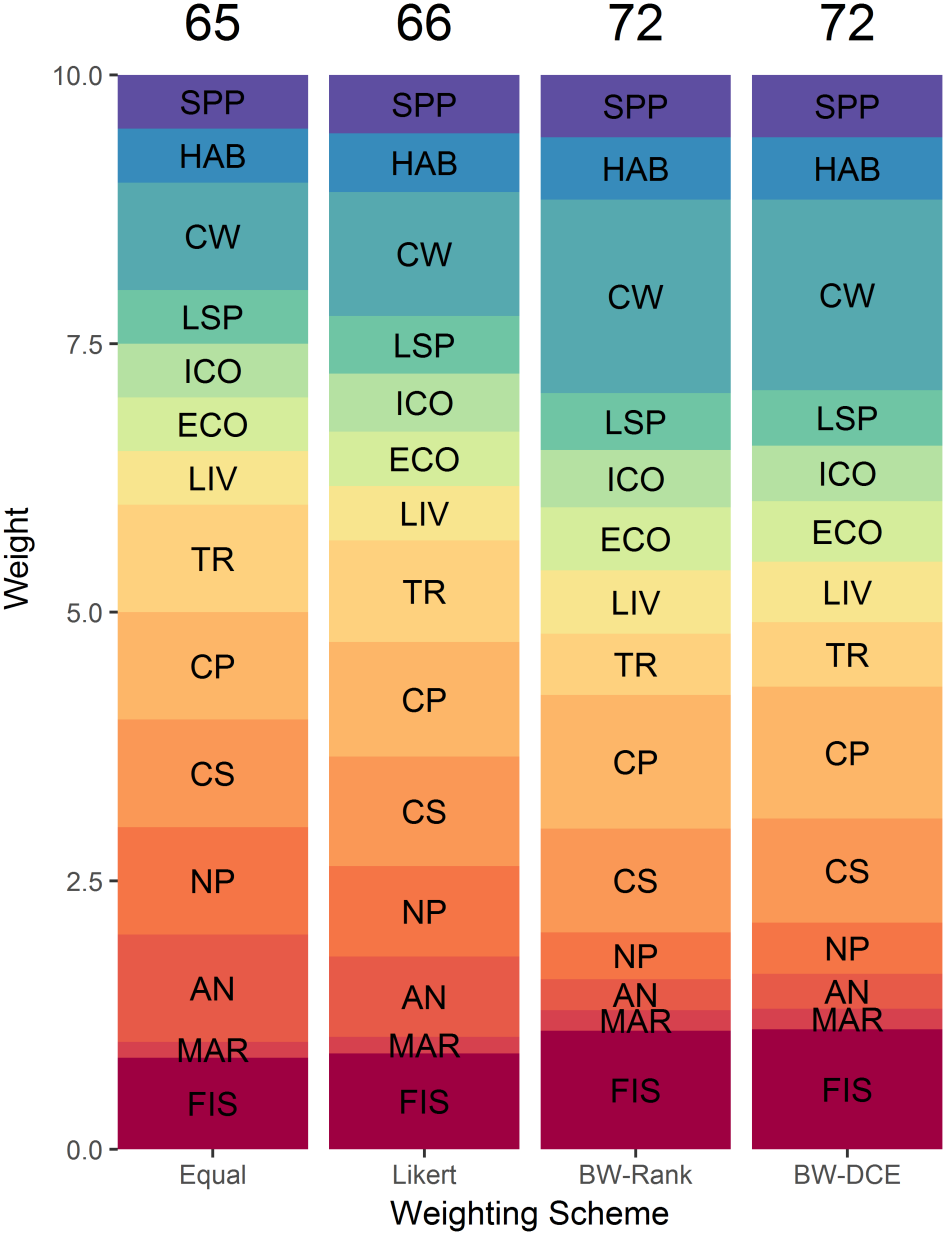
Four weighting schemes for combining constituent goals or subgoals of the Canadian Ocean Health Index. Weights are represented as the height of each band, and the overall Index score is indicated at the top of each weighting scheme. ‘Equal’: all goal weights = 1; ‘Likert’: weights are derived from Likert-scale survey questions; ‘BW-Rank’ and ‘BW-DCE’: weights are derived from the answers of best/worst survey questions. See Methods for details. Goal and subgoal codes are as in Fig 3.

Applying unequal goal weightings based on the opinion of the Canadian public increased COHI scores (Figs 3 and 4). The Likert-derived weighting resulted in a COHI score closest to that of the COHI with equal weighting, and all Likert goal weights are within ± 0.25 of 1 (Fig 4). While both weighting methods derived from the best/worst questions resulted in the same overall COHI score after rounding, goal-specific weights were more variable than the Likert-derived weights, being within ± 0.77 (BW-Rank) and ± 0.80 (BW-DCE) of 1 (Fig 4).

**Fig 5:**
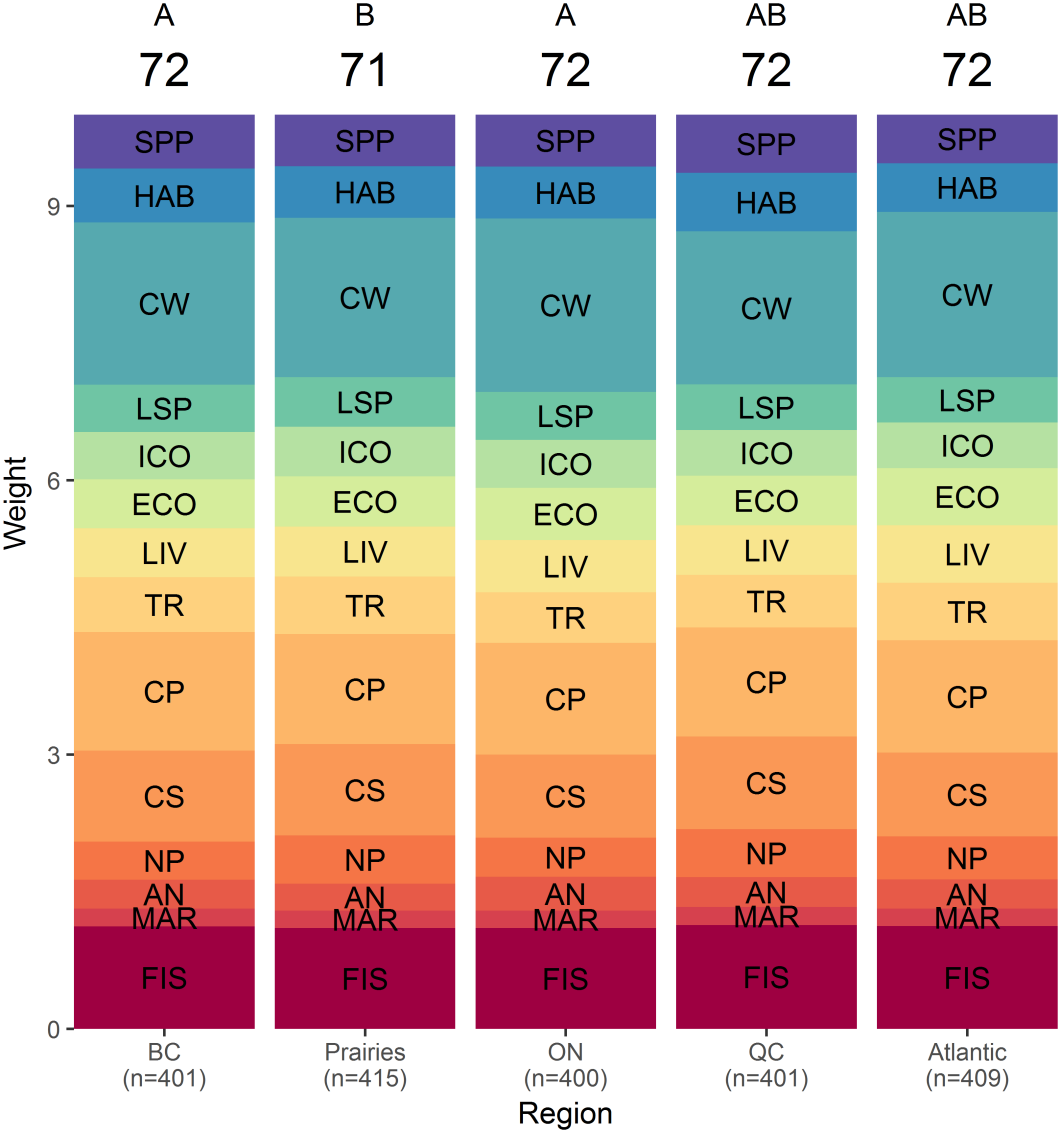
BW-DCE weighing across five regions for constituent goals or subgoals of the Canadian Ocean Health Index. Weights are represented as the height of each band, as estimated by discrete choice model coefficients (see Methods for details), and the overall Index score is indicated at the top of each region. Similar letters above the overall Index indicate that the indices differed in less <5% of simulated cases. BC: British Columbia, Prairies: Alberta, Manitoba, and Saskatchewan, ON: Ontario, QC: Quebec, Atlantic: New Brunswick, Nova Scotia, Prince Edward Island, and Newfoundland. Goal and subgoal codes are as in Fig 3.

**Fig 6:**
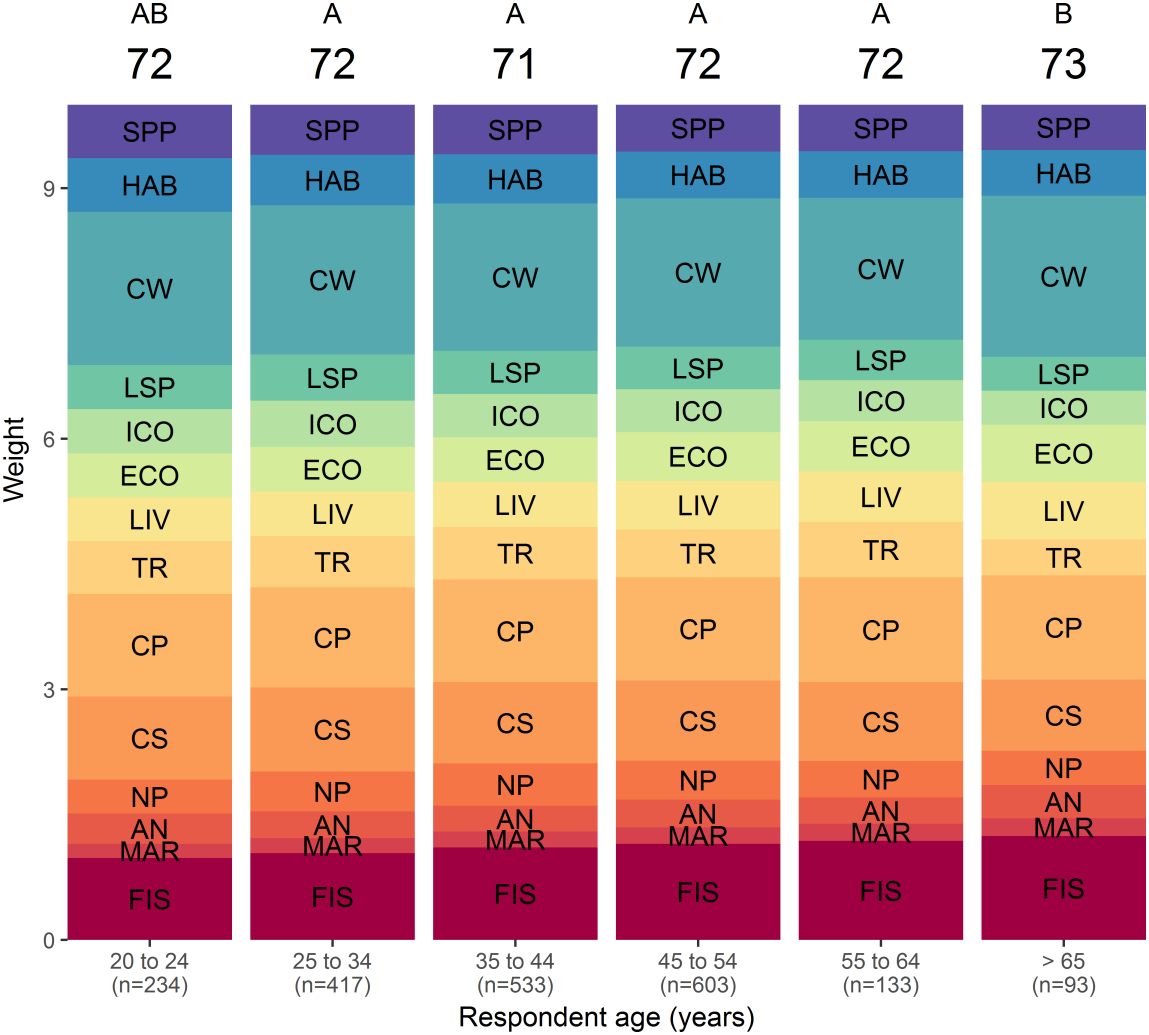
BW-DCE weighing across six age groups for constituent goals or subgoals of the Canadian Ocean Health Index. Weights are represented as the height of each band, as estimated by discrete choice model coefficients (see Methods for details), and the overall Index score is indicated at the top of each age group. Similar letters above the overall Index indicate that the indices differed in less <5% of simulated cases. Goal and subgoal codes are as in Fig 3.

**Fig 7:**
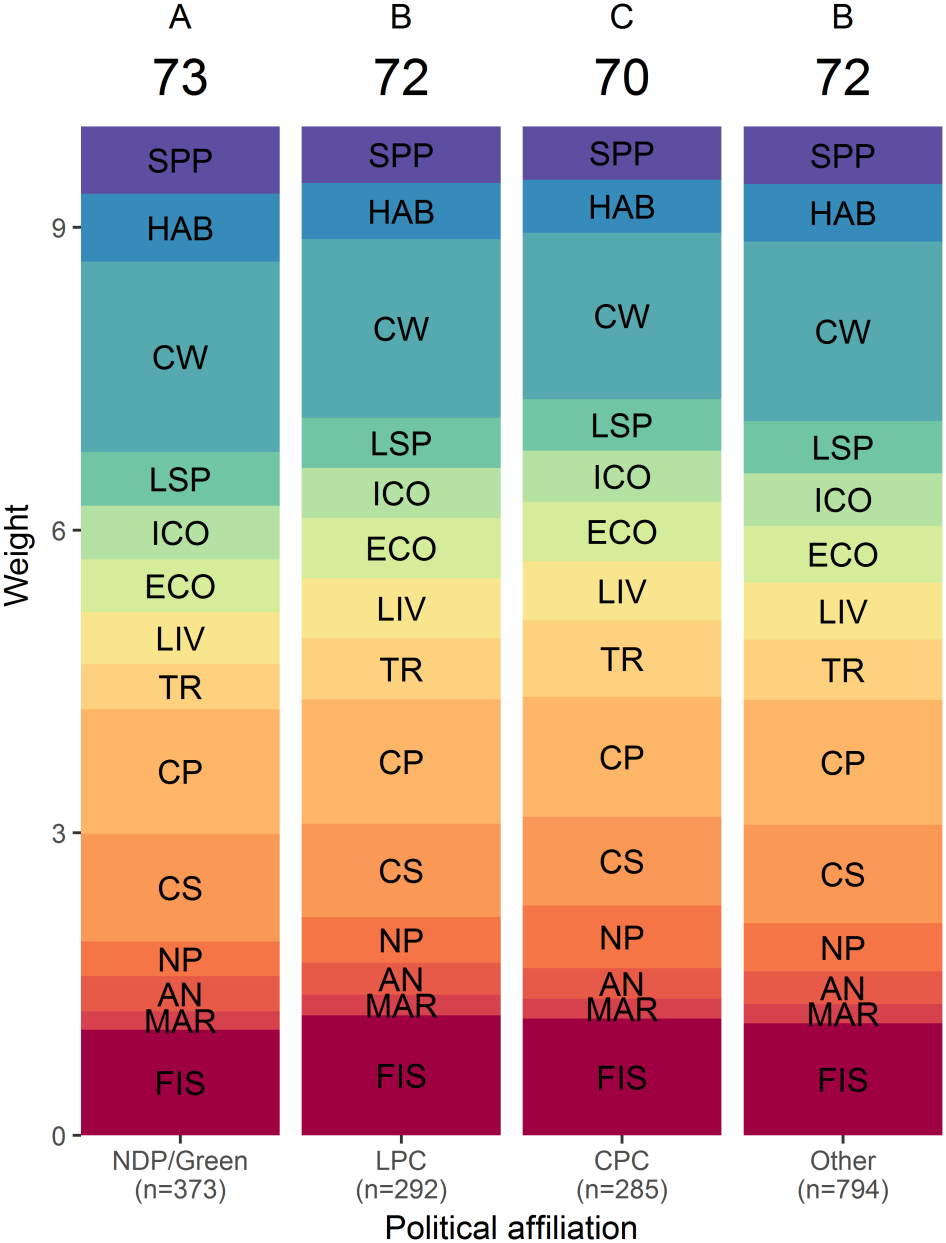
BW-DCE weighing across four political affiliations for constituent goals or subgoals of the Canadian Ocean Health Index. Weights are represented as the height of each band, as estimated by discrete choice model coefficients (see Methods for details), and the overall Index score is indicated at the top of each political affiliation. Similar letters above the overall Index indicate that the indices differed in less <5% of simulated cases. NDP/Green: New Democratic Party & Green Party; LPC: Liberal Party of Canada; CPC: Conservative Party of Canada. Goal and subgoal codes are as in Fig 3.

**Fig 8:**
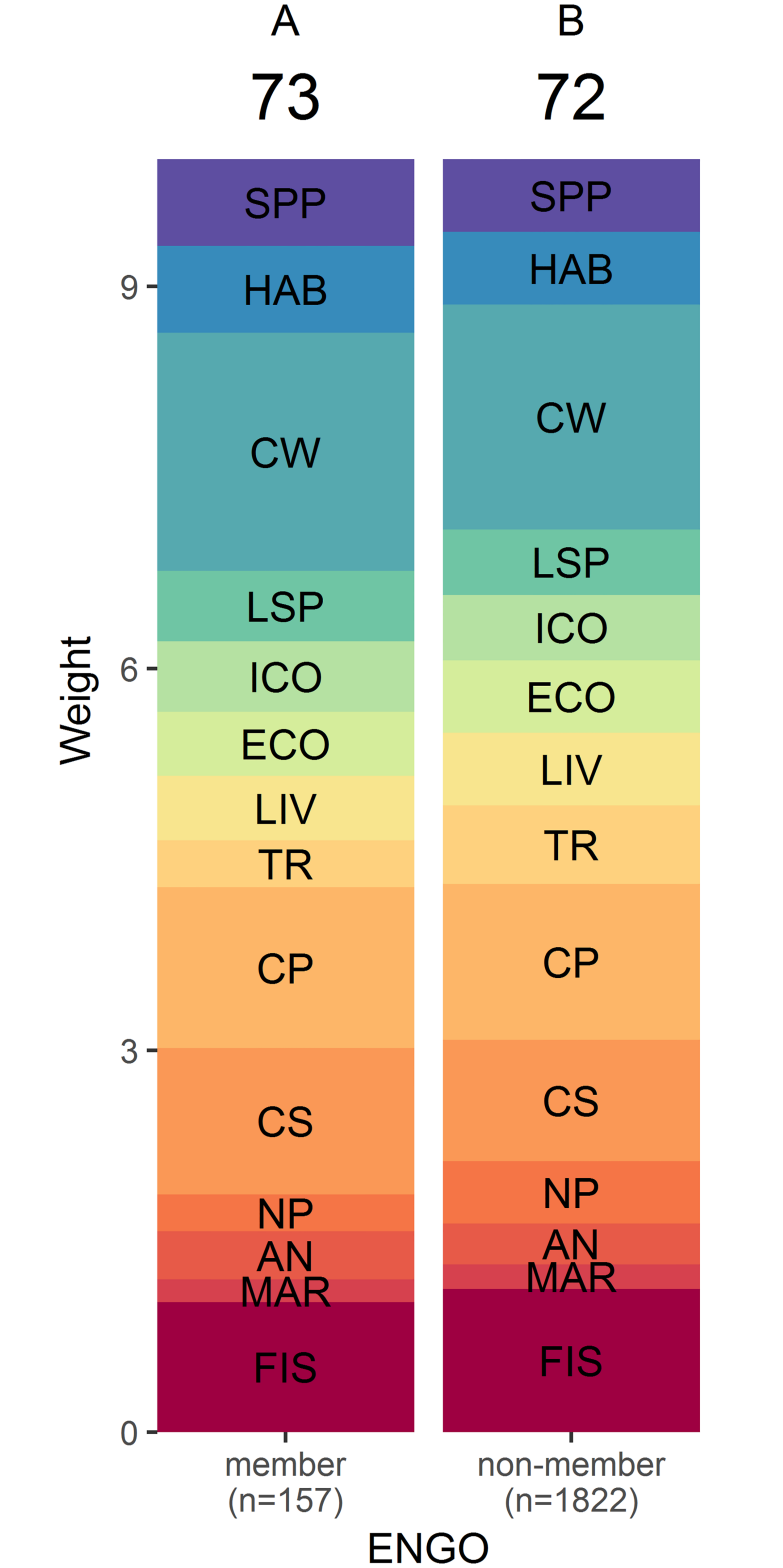
BW-DCE weighing of whether or not respondents are members of environmental non-governmental organisations for constituent goals or subgoals of the Canadian Ocean Health Index. Weights are represented as the height of each band, as estimated by discrete choice model coefficients (see Methods for details), and the overall Index score is indicated at the top of each category. Similar letters above the overall Index indicate that the indices differed in less <5% of simulated cases. Goal and subgoal codes are as in Fig 3.

The COHI scores obtained with BW-DCE weights calculated from respondent answers grouped by region (Fig 5), age (Fig 6), political affiliation (Fig 7) and environmental engagement (Fig 8) were nearly uniform (i.e., respondents across levels of each factor yielded similar average weights). The COHI score for the Prairies region was 1 point lower than the other regions (Fig 5). The weight for Clean Waters was higher in Ontario, and lower in Quebec. The lowest weight for Carbon Storage was observed in Ontario. There was a two-point spread in COHI scores across age groups (Fig 6). With increasing age, the weight assigned to Food Provision increased and that of Biodiversity decreased. Similarly, there was a two-point difference in COHI scores among respondents with very different political affiliations (Fig 7). The greatest difference was among those affiliated with the NDP/Green parties, who assigned a higher importance to Biodiversity, Clean Waters, and Carbon Storage, and respondents identifying with the CPC, who assigned a higher importance to Livelihoods & Economies, Tourism & Recreation, and Natural Products. Whether respondents were environmentally engaged (i.e., by being members of environmental organizations) or not changed the COHI score by only 1 point, even though there were considerable differences between groups in the importance accorded to Biodiversity, Carbon Storage, and Tourism & Recreation (Fig 8).

## Discussion

The Ocean Health Index (OHI) aims to provide an intuitive means to understand and track the condition of ocean services globally. It can also be a useful management tool when applied at national or subnational scales, to match the scale of conservation planning and interventions. National governments of Colombia and China are examples of jurisdictions already using the OHI framework to identify gaps in data collection and standardize information in databases for interpretation and use in independently-led OHI assessments [12]. We have provided a first customized national OHI score for Canada (COHI), redefining some goals and incorporating new data sources as previous sub-global assessments have done (e.g., [13–15]). More importantly, we devised and tested new schemes to weight individual OHI goals to reflect broadly held public preferences. Weighting OHI goals resulted in considerably higher OHI scores (1–7 points) than an unweighted approach, regardless of the weighting method used. However, the weights derived from a best-worst choice experiment provided more divergent importance scores (ranges of 0.75 to 1.15, and 0.33 to 1.77 for the Likert and BW-DCE schemes, respectively), and hence a clearer distinction, among goals than a simple Likert-scale survey [17]. Because discrete choice experiments force respondents to make trade-offs among goals (i.e., not all goals in a set can be rated as being very important), the scores they generate are a more accurate reflection of what really matters to people [40,41]. Adopting a weighting scheme based on coefficients from the best-worst discrete-choice experiment model increased the Canadian Ocean Health Index (COHI) score compared to an unweighted scheme by 7 points. This effect was greater than the 5 point decrease caused by changes to data and definitions, highlighting the importance of devising meaningful goal weights to enhance the national or regional relevance of the Ocean Health Index. It is important to note that using region-specific goal weights can also lower overall Index scores, depending on the individual goal scores and which goals people value most. Such changes, whether they increase or decrease overall scores, are appropriate and more accurate to how people perceive the health of the ocean.

Customizing the OHI to reflect the Canadian context required that at least one goal considered explicitly the access of Aboriginal people to traditional marine resources. We did so by redefining the original ‘Artisanal Fishing Opportunities’ goal into a Canadian ‘Aboriginal Needs’ goal. This change had the largest single impact on goal scores of all the data modifications we implemented. Although necessary to represent Canada more holistically, the new ‘Aboriginal Needs’ goal presents two minor drawbacks. First, our focus on Aboriginal people means that this goal now overlooks the need and access of non-Aboriginal Canadians to subsistence fishing and hunting opportunities. However, in Canada these subsistence activities are part of Aboriginal cultural identity and are prioritized over other recreational and commercial fisheries [38,42]. Additionally, the consumption of fish by coastal Aboriginal communities is almost four times higher than the overall (for all groups) Canadian average [43]. Second, the inputs for calculating ‘Aboriginal Needs’ may have to be modified to allow the calculation of sub-national COHI scores, e.g. for each of Canada’s three delimiting oceans. For example, one of the driving variables of AN is relative ice cover because ice allows winter travel by Inuit and First Nations hunters and fishermen in the North [37,38], but Aboriginal communities around the Bay of Fundy (Atlantic) and much of BC (Pacific) experience largely ice-free winters. Climate change will still constrain access to traditional ocean resources in these more southern communities through changes in the availability and behaviour of target species, incidence of disease, and risk of food spoilage [38], but the exact temperature-related proxy for access to resources might be more difficult to identify and measure than ice cover. The other components of AN, namely fuel and food prices, are likely to be relevant to all Aboriginal communities. We recommend undertaking direct consultation with Aboriginal groups to improve the regional relevance of the AN goal at smaller spatial scales. However, it is important to remember that drastic regional customization of the data inputs will make AN goal scores, and more generally COHI values, not comparable across regions.

The survey, described further in Daigle *et al*. [17], used to derive the weights used in this manuscript did not include respondents from the norther territories. Consequently, the weightings in this manuscript do not reflect the opinions of those northern populations. However, 97% of Canadian Aboriginals reside in the provinces sampled by the survey and the total population of the northern territories represent 0.3% of the total Canadian population.

Changes in data sources to reflect the Canadian context had variable effects on specific goal scores and on the overall COHI scores. Inclusion of the AN goal instead of the original ‘Artisanal Fishing Opportunities’ goal decreased substantially the unweighted COHI score (by 6 points) compared to the similarly unweighted OHI score for Canada in the global analysis [8]. This difference is largely explained by the declining trend in AN scores, driven mainly by increasing fuel prices and, to a lesser extent, by shrinking ice cover. Incorporating additional iconic species and new carbon storage habitats made up for some (2 points), but not all, of this loss in unweighted COHI score. The score of the Iconic Species subgoal increased when we expanded the list of iconic species because several of the added species are at lower risk of extinction than the species in the original OHI list. The score of the revised Carbon Storage goal also increased compared to that in the original OHI because we added large areas of carbon-rich habitats (i.e., subsea permafrost and methane clathrate fields) prevalent in the Canadian North but omitted from the original OHI, which are currently in relatively good ‘health’. The capacity of these habitats of store carbon is, however, under threat from warming sea temperatures [20,33,35], hence future declines in the Canadian Carbon Storage score should be expected.

People do not value the various goals of the Ocean Health index equally. For example, the Clean Waters goal appears to be the most important of all goals to panels of US experts and stakeholders [16] and to the general public in Canada [17]. While there is some geographic variability in societal values among Canadians, all regions were surprisingly homogenous in their priorities. Surprisingly, goals that directly benefit those living on the coast (e.g., Coastal Protection) were not prioritized by respondents from coastal regions. Politically left-leaning respondents displayed a preference for non-extractive, publicly shared benefits (e.g. Biodiversity, Carbon Storage), while right-leaning respondents were more likely to prefer extractive, individual benefits, that represent a more direct and immediate financial incentive (e.g. Livelihoods and Economies, Tourism & Recreation, Natural Products). More details on the goal preferences of social, political, and demographic groups can be found in Daigle *et al*. [17].

The OHI should arguably reflect societal values, which can be incorporated as goal weights in calculation of the index [8]. We quantified preferences of the general public using Likert scores, simple ranks from a best-worst choice experiment, and model coefficients from the same choice experiment. The best-worst discrete choice experiment (BW-DCE) provided the clearest discrimination among goals. While Likert-scale questions are simpler to distribute and analyse, the answers lack the implicit trade-offs that respondents must make in best/worst choice experiments, which force them to reveal only their extreme preferences. In natural resource management, and other field where the resources and budgets are limited, the Likert scale is less useful for prioritizing attributes since it does not force respondents to operate within a limited scope (i.e. respondents can chose to answer ‘most important’ for all 10 goals) Best/worst choice experiments are therefore advocated when trying to rank many attributes (or goals, in this case) because the method avoids the problem of respondents discriminating poorly among attributes of intermediate importance [40,41]. Likewise, discrimination power is lost by simply ranking the answers from best/worst choice experiments (BW-Rank). The BW-DCE also has the advantage of creating a statistical model with associated error terms, and can also deal with an unbalanced design in terms of question sets for best/worst questioning unlike BW-Rank. Consequently, we suggest using the coefficients from models fitted to best/worst answers as weighting scheme in OHI calculations since these coefficients most accurately reflect both public opinion and the trade-offs faced by policy-makers. More generally, we recommend that opinion about goal importance be elicited from the general public to avoid potential stakeholder or expert bias [44–47].

We view the development of a Canadian Ocean Health Index as an important step for ocean conservation in Canada. The OHI provides a framework integrating 10 important goals that relate to ocean health, and has been incrementally improved since its first publication [8] through annual global assessments [10] and independently-led assessments [12]. This initial iteration of the COHI can be used as a baseline against which future COHI scores can be compared and as a means to predict likely public support for management actions when weights based on public priorities are used. By building on this work, the COHI can act as a management tool to prioritise actions on a national scale and as an intuitive means of communicating the complex notion of ocean health to a broad audience. However, given that Canada borders three oceans that are distinct ecologically, socially, and economically, the need for sub-national (i.e., ocean-specific) assessments is evident. The customized COHI will facilitate this task greatly.
